# Underexpression of LKB1 tumor suppressor is associated with enhanced Wnt signaling and malignant characteristics of human intrahepatic cholangiocarcinoma

**DOI:** 10.18632/oncotarget.4305

**Published:** 2015-05-27

**Authors:** Jinghan Wang, Keqiang Zhang, Jinhui Wang, Xiwei Wu, Xiyong Liu, Bin Li, Yan Zhu, Yong Yu, Qingbao Cheng, Zhenli Hu, Chao Guo, Shuya Hu, Bing Mu, Chun-Hao Tsai, Jie Li, Lynne Smith, Lu Yang, Qi Liu, Peiguo Chu, Vincent Chang, Baihe Zhang, Mengchao Wu, Xiaoqing Jiang, Yun Yen

**Affiliations:** ^1^ The First Department of Biliary Surgery, Eastern Hepatobiliary Surgical Hospital, The Second Military Medical University, Shanghai, China; ^2^ Department of Molecular Pharmacology, City of Hope National Medical Center, Duarte, California, USA; ^3^ The Integrative Genomics Core lab of Department of Molecular Medicine, City of Hope National Medical Center, Duarte, California, USA; ^4^ Changhai Hospital, The Second Military Medical University, Shanghai, China; ^5^ Department of Orthopedic Surgery, School of Medicine, China Medical University, Taichung, Taiwan; ^6^ Department of Pathology, City of Hope National Medical Center; Duarte, California, USA; ^7^ Program for Translation Medicine, Taipei Medical University, Taipei, Taiwan; ^8^ PhD Program of Cancer Biology and Drug Discovery, College of Medical Science and Technology, Taipei Medical University, Taipei, Taiwan

**Keywords:** intrahepatic cholangiocarcinoma (ICC), liver kinase B1 (LKB1), global transcriptional profiling, Wnt/β-catenin, recurrence and metastasis

## Abstract

Intrahepatic cholangiocarcinoma (ICC) is a rare and highly aggressive malignancy. In this study, we identified the presence of gene deletion and missense mutation leading to inactivation or underexpression of liver kinase B1 (LKB1) tumor suppressor and excluded the involvement of LKB1 gene hypermethylation in ICC tissues. Immunohistochemical analysis showed that LKB1 was underexpressed in a portion of 326 ICC tissues compared to their adjacent normal tissues. By statistical analysis underexpression of LKB1 in ICC tissues significantly correlated with poor survival and malignant disease characteristics in ICC patients. Moreover, we showed that knockdown of *LKB1* significantly enhanced growth, migration, and invasion of three LKB1-competent ICC cell lines. Global transcriptional profiling analysis identified multiple malignancy-promoting genes, such as *HIF-1α*, *CD24*, *Talin1*, *Vinculin*, *Wnt5*, and signaling pathways including Hedgehog, Wnt/β-catenin, and cell adhesion as novel targets of LKB1 underexpression in ICC cells. Furthermore, knockdown of LKB1 gene expression dramatically enhanced Wnt/β-catenin signaling in ICC cells, while an inverse correlation between LKB1 and nuclear β-catenin was observed in ICC tissues. Our findings suggest a novel mechanism for ICC carcinogenesis in which LKB1 underexpression enhances multiple signaling pathways including Wnt/β-catenin to promote disease progression.

## INTRODUCTION

Intrahepatic cholangiocarcinoma (ICC) is the second most common hepatic malignancy and is highly aggressive [1, 2]. Epidemiologic data indicate that the incidence and mortality of ICC has risen worldwide in recent decades [1, 2]. Patients with ICC typically present at advanced stages, and the vast majority of patients with unresectable disease die between 6 to 12 months following diagnosis [1, 2]. The lack of effective therapeutic strategies may be attributed in part because the underlying molecular mechanisms of the disease are poorly understood. Therefore, a better understanding of the molecular biological features may facilitate novel diagnostic and therapeutic approaches for ICC patients.

Recent studies suggest that the molecular pathogenesis of ICC may have similarities with the pathogenesis of HCC because both diseases share dominant risk factors, such as cirrhosis, HBV, HCV and metabolic syndrome [3]. Genetic alterations in several oncogenes and tumor suppressor genes have been identified in ICC. Specifically, *P53* mutations occur in ∼21% of ICC, and activating *K-RAS* mutations are frequently detected in ICC [4]. Mutations in other genes, including *EGFR, N-RAS, PI3K*, and *APC*, have been less frequently described [5]. In ICC patients several intracellular signaling pathways such as EGF, HGF/MET, VEGF and K-RAS/MAPK or IL-6/STAT are deregulated [6]. Several clinical studies have also demonstrated the aberrant activation of Wnt/catenin signaling in ICC [7, 8]. Most recently, a study has discovered frequent inactivating mutations in multiple chromatin-remodeling genes in ICC [9], and another study has also revealed recurrent novel FGFR2 fusions and ERRFI mutations in ICC [10].

Liver kinase B1 (*LKB1*) is a serine/threonine kinase (also named *STK11*) which plays essential roles in development and cell polarity [11]. It was first recognized as a tumor suppressor gene from germ-line mutations that cause Peutz–Jeghers syndrome, which is characterized by gastrointestinal hamartoma with an increased risk of various cancers [12]. Inactivating mutations in *LKB1* are also found in sporadic lung adenocarcinoma, cervical, breast, and pancreatic cancers [13-15]. The LKB1 master kinase activates adenosine monophosphate-activated protein kinase (AMPK) and 12 other similar kinases, to regulate cell polarity, metabolism, and growth [16, 17]. LKB1 deficiency can significantly enhance the carcinogenic effect of *P53* or *PTEN* inactivation, or of *K-RAS* activation in mouse models [18-20]. An important role for LKB1 in cancer metastasis was demonstrated when inactivation of LKB1 significantly enhanced metastasis of lung adenocarcinomas and melanoma driven by oncogenic *K-RAS* [21, 22]. It has recently been reported that inactivation of LKB1 promotes metabolic reprogramming of cancer cells via HIF-1α to enhance their growth and survival under low-nutrient conditions [23].

To date, *LKB1* genetic alterations and protein expression have not been characterized in ICC patients. Therefore, we investigated the status and potential roles of LKB1 in a large ICC cohort. In the study, we demonstrate that LKB1 protein is inversely associated with malignancy and poor survival of ICC patients. We further uncovered potential target genes and critical signaling pathways such as Wnt/β-catenin and cell adhesion that are affected by *LKB1* knockdown in ICC cell lines. We investigated the cross-talk between the Wnt/β-catenin pathway, which in ICC is aberrantly activated in the absence of APC and E-Cad mutations [7, 8], and LKB1 dysregulation in ICC cells using LKB1 knockdown. We also discovered an inverse correlation between LKB1 and nuclear β-catenin in our ICC cohort. This suggests that *LKB1* underexpression may partially enhance activation of the WNT/β-catenin pathway and thus contribute to the malignancy and progression of ICC.

## RESULTS

### Identification of genetic alterations and methylation status of LKB1 gene in ICC tissues

**Figure 1:**
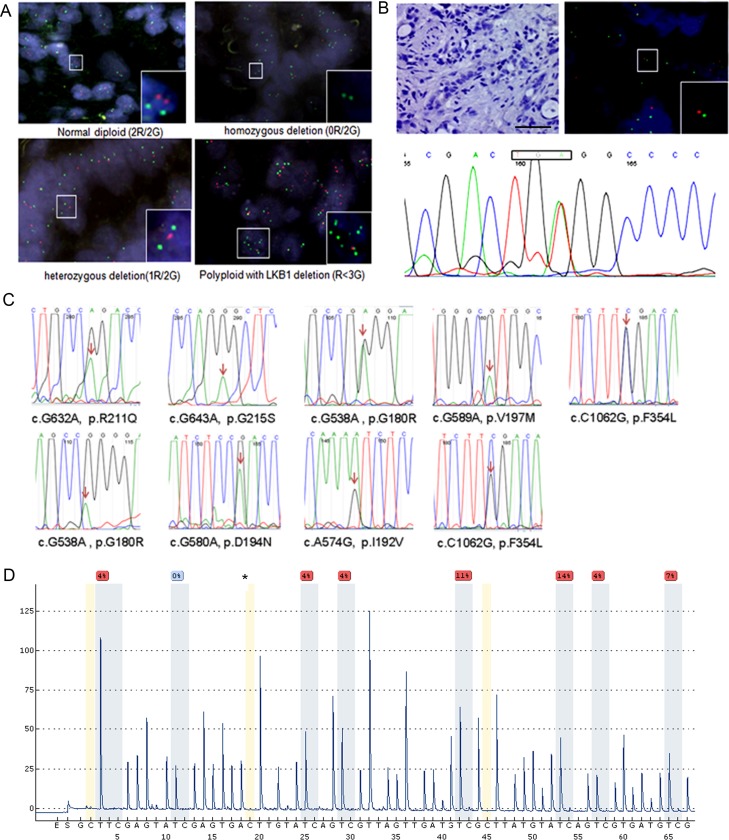
Genetic alterations and methylation of LKB1 in cholangiocarcinomas **A.** Dual-color fluorescence in situ hybridization (FISH) of cholangiocarcinoma tissues as indicated where green indicates the reference of chromosome 19, and red the *LKB1* locus. Upper left: normal diploid (2Red/2Green), upper right: homozygous deletion (0R/2G), lower left: hemizygous deletion (1R/2G) and lower right: polypoid (3G>R). **B.** Illustrates a second genetic alteration in a representative cholangiocarcinoma tissue with heterozygous *LKB1* deletion. Tumor section negative for LKB1 by IHC (x200, upper-left), LKB1-FISH (upper-right), T834A nonsense (lower panel) mutation of exon-6 resulting in a premature stop codon, scale bar: 20 μM. **C.** Sanger sequencing maps of 9 missense mutations identified in 147 ICC tissue samples. The heterozygous mutated nucleotide was labeled by arrows in the map. **D.** Measurement of the LKB1 methylation level using pyrosequencing in a representative ICC tissue. The asterisks indicate no residual C at the non-CpG site, ensuring complete bisulfite conversion.

We first measured the genetic alteration of *LKB1* in a total of 288 ICC samples using molecular approaches. By fluorescence in situ hybridization analysis homozygous and heterozygous deletions of *LKB1* were found in 2.1% (6/288) and 2.4% (7/288) of ICC tissues. Interestingly, a deletion of LKB1 with aneuploidy of chromosome 19 was also found with low frequency in ICC tissues (Figure 1A). We further examined the genetic alterations leading to the complete inactivation of *LKB1* in tissues with heterozygous *LKB1* deletion by exon sequencing. We found a genetic alteration leading to complete inactivation of LKB1 in one case. As shown in Figure 1B, IHC showed loss of LKB1, while FISH displayed a heterozygous *LKB1* deletion in the ICC tumor area, and sequencing identified a nonsense mutation. Using Sanger sequencing, from a total of 147 ICC samples we identified 6 novel heterozygous missense mutations and one case of the previously reported c.G580A, p.D194N and two cases of the well-characterized loss-of-function mutation of c.C1062G, p.F354L (Figure 1C), which frequently occurred in lung carcinomas [24]. Loss-of-function of novel missense mutations should be further validated by functional assays. Additionally, we examined the methylation level of LKB1 in 21 ICC tissues and 12 non-malignant tissues using pyrosequencing analysis. Results of a CpG methylation analysis of the representative ICC and adjacent non-malignant tissues are presented in Figure 1D and Supplementary Figure 2. ICC tissues showed moderate levels of LKB1 methylation that were significantly higher compared with non-malignant tissues (*P* < 0.05, Supplementary Table 2).

### Underexpression of *LKB1* predicts aggressive clinicopathological characteristics and poor prognosis in ICC patients

**Figure 2:**
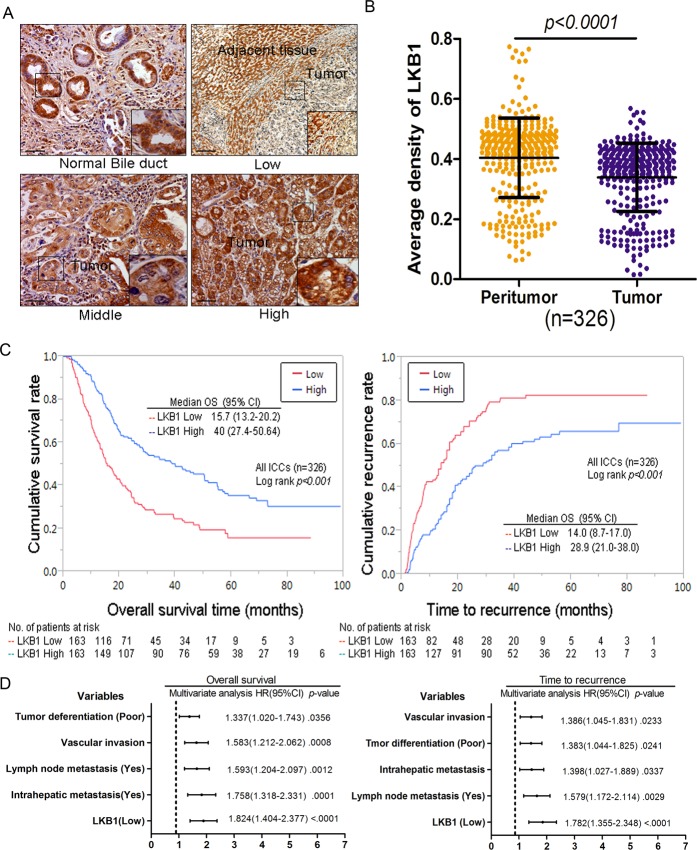
Decreased expression of LKB1 correlates with poor prognosis in cholangiocarcinoma patients **A.** Photomicrographs of three representative cholangiocarcinoma sections stained for high, intermediate, and low LKB1 expression ( x 200), scale bar: 20 μM. **B.** Comparison of relative IHC staining of LKB1 expression in paired ICC tissue samples (*N* = 326). **C.** Kaplan-Meier analysis for OS and TTR of patients with different LKB1 expression levels. **D.** Cox multivariate proportional hazard regression analyses for OS and TTR of 326 ICCs. The variables included in the multivariate analysis were selected by univariate analysis.

We further examined LKB1 protein in 326 ICC samples by IHC analysis. Representative LKB1 immunostaining of ICC tissues are shown in Figure 2A, and reveal a mainly cytoplasmic staining pattern in both tumor and peritumoral tissues. IHC analysis of LKB1 protein showed that LKB1 was significantly under-expressed in a high percentage of ICC tissues compared with the matched peritumor bile duct tissues (mean LKB1 density 0.326 ± 0.021 vs. 0.369 ± 0.027, respectively, *P* < 0.05) as shown in Figure 2B. Quantitative analysis of IHC revealed that LKB1 protein was decreased by at least 50% in 23.6% (77/326) of ICC tissues. We found that the level of LKB1 expression was significantly correlated to several aggressive clinicopathological characteristics, such as poor differentiation (*P* < 0.001), advanced stage tumor (TNM stages III-IVA; *p* < 0.001) and tumor recurrence (*P* < 0.001) (Table 1).

**Table 1:** The correlation between clinicopathologic characteristics and LKB1 expression in ICC tissues

	LKB1 staining		
Parameter	Low	High	*P* value
Age			
≤58 years old	97	59	
>58 years old	90	80	0.0920
Gender			
Male	122	93	
Female	65	46	0.7536
Alcohol intake			
Yes	97	80	
No	90	59	0.3084
CA19-9 level			
≤37 (U/ml)	76	59	
>37 (U/ml)	111	80	0.7436
Virus infection			
HBV	68	85	
HCV	8	11	
HBV+HCV	4	4	
None	106	40	0.5796
Liver cirrhosis			
No	151	101	
Yes	36	38	0.0847
Tumor size			
≤5cm	100	51	
>5cm	112	63	0.6743
Differentiation			
Poor	55	16	
Moderate	107	74	
Well	25	49	<0.001
Vascular invasion			
No	113	89	
Yes	74	50	0.5078
Lymph node metastasis			
No	111	97	
Yes	76	42	0.0527
Encapsulation			
Complete	18	18	
None	169	121	0.3436
Intrahepatic metastasis			
No	130	109	
Yes	57	30	0.0724
TNM stage b			
I-II	70	83	
III-IVA	117	56	<0.0001
Recurrence			
No	54	60	
Yes	133	79	0.0075

Note: The χ^2^ test was used for comparison between groups.

Survival analysis was performed to investigate the clinical signiﬁcance of LKB1 expression in the progression of ICC. All 326 ICC patients were divided into 2 groups: high LKB1 expression (*N* = 163) and low LKB1 expression (*N* = 163). The Log-rank test indicated that low expression of LKB1 is significantly related to poorer OS (15.7 months, vs. 40.0 months for high levels; *p* < 0.001) and shorter TTR (14.0 months, vs. 28.9 months for high levels; *p* < 0.001) as shown in Figure 2C. To avoid confounder effects, multivariate COX analysis was conducted on this ICC population. Low expression of LKB1 is an independent risk factor for both OS (HR = 1.824; 95% CI: 0.404–2.377; *p* < 0.001) and TTR (HR = 1.782: 95% CI: 1.355–2.348; *p* < 0.001) for ICC patients (Figure 2D). Here we also found tumor differentiation, vascular invasion, lymph node metastasis and intrahepatic metastasis showed similar predictive power to OS and TTR (Figure 2D).

### LKB1 downregulation promotes growth, migration, and invasion of ICC cells

**Figure 3:**
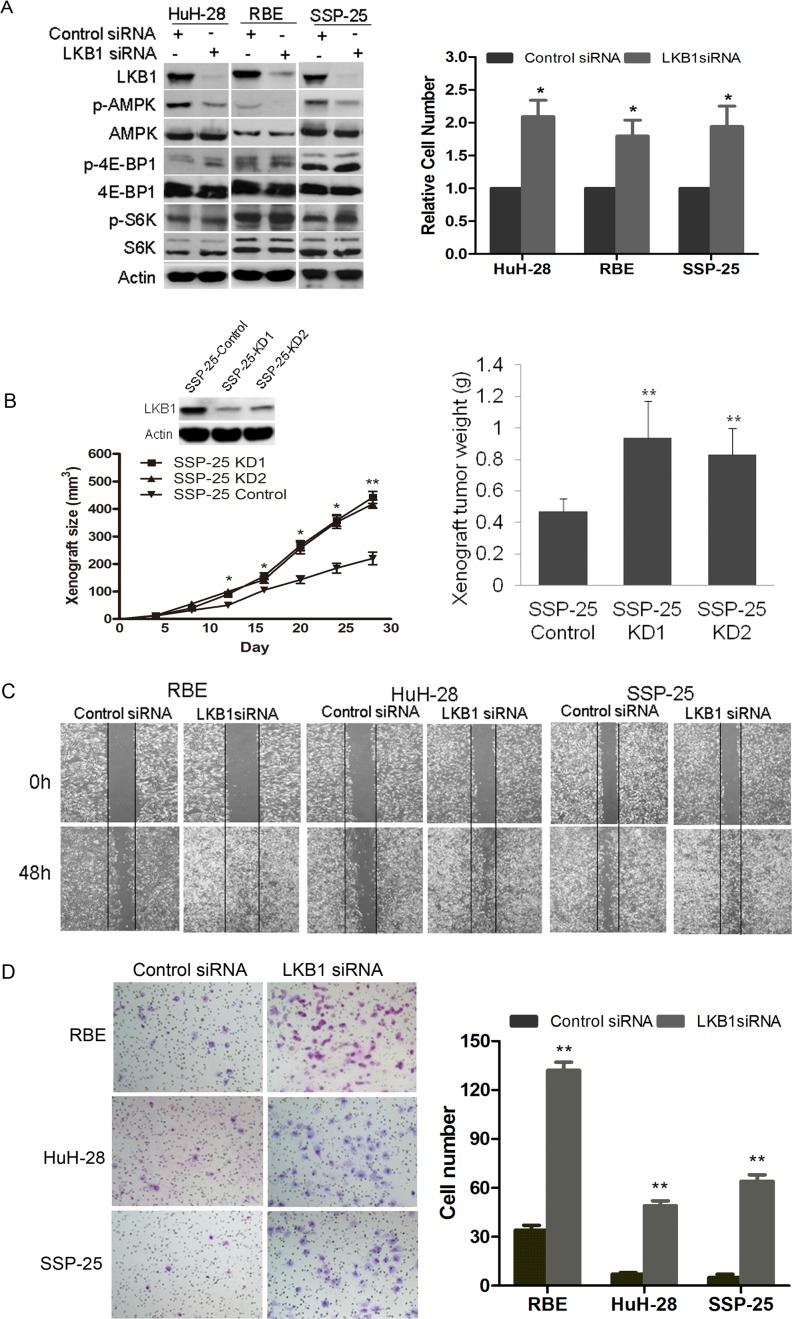
Impact of knockdown of LKB1 on growth, migration and invasion of ICC cell lines **A.** Western blot analysis of cell lysates of HuH-28 and RBE cells transfected with either LKB1 siRNA or scramble (control) siRNA harvested at 72 h post transfection. Actin serves as loading control, and phosphorylated and total AMPK, 4E-BP1, and S6K were detected with the appropriate antibodies. Effective knockdown of LKB1 in both HuH-28 and RBE cells was verified (left panel). In the right hand panel a histogram of 72h proliferation shows knockdown of LKB1 increased the *in vitro* proliferation of HuH-28, RBE and SSP-25 cells (*P < 0.05, compared to control siRNA). **B.** Downregulation of LKB1 promoted the growth of SSP-25 *in vivo*. Western blot of LKB1 in SSP-25-Control and SSP-25-KD1/2 (control for control shRNA transfected, KD1/2 for LKB1 shRNA1/2 transfected; upper-left); tumor growth curves of SSP-25-Control and SSP-25-KD1/2 (lower-left); quantitative analysis of weight (g) of tumor xenografts (right, **P < 0.01, compared to control shRNA). **C.** Representative photomicrographs of the wound healing assay at the times indicated in scramble (control) and LKB1 siRNA transfected cells as shown. Vertical black lines indicate the initial extent of the clearing of the cell monolayers. **D.** Lower surface of Matrigel transwell membranes seeded ICC cells transfected with siRNAs as indicated after 24 h incubation is shown on the left with quantitative analysis of invaded cells shown on the right. Data are shown as mean values graphed for indicated cells on the right (*N* = 3 replicates per cell type). Error bars show SD ((*P < 0.05, ^**^P < 0.01, compared with control siRNA).

To explore the impact of LKB1 on cellular features of malignancy including proliferation, migration, and invasiveness of ICC, we attenuated LKB1 expression in three human ICC cell lines HuH-28, RBE and SSP-25. LKB1 knockdown (Figure 3A, left panel) resulted in a significant increase of *in vitro* cellular proliferation in RBE and HuH-28 and SSP-25 cells at 72h post-transfection of LKB1 siRNA (Figure 3A, right panel). Loss of LKB1 may cause inactivation of AMPK and derepression of mTOR protein kinase [25]. We observed a significant decrease in AMPK phosphorylation but a slight increase in phosphorylation of two mTOR downstream signaling proteins S6K and 4E-BP1 in LKB1-attenuated ICC cells (Figure 3A, left panel). To assess the effect of LKB1 on tumour growth *in vivo*, we constructed SSP-25-KD1/2 cell lines, which stably express *LKB1* specific shRNA1/2 to specifically decrease *LKB1* expression (Figure 3B, upper-left). Compared to the SSP-25-Control (expressing scramble shRNA), SSP-25-KD1/2 (LKB1 knockdown) xenografts grew significantly faster, beginning ∼12 days after transplantation of tumour cells (Figure 3B, lower-left, *p* < 0.05). Correspondingly, the average weights of SSP-25-KD1/2 xenografts were also significantly higher in comparison to SSP-25-Control xenografts (Figure 3B, right, *p* < 0.01). We further studied the effects of LKB1 on the *in vitro* migration and invasion of LKB1-competent ICC cells. A wound-healing assay showed that knockdown of LKB1 greatly increased the migration potential of HuH-28, RBE, and SSP-25 cells (Figure 3C). Likewise, knockdown of LKB1 significantly enhanced the invasive potential of all three cell lines in the Matrigel invasion assay (Figure 3D, *p* < 0.05).

### Enriched gene sets in LKB1-attenuated ICC cells

**Figure 4:**
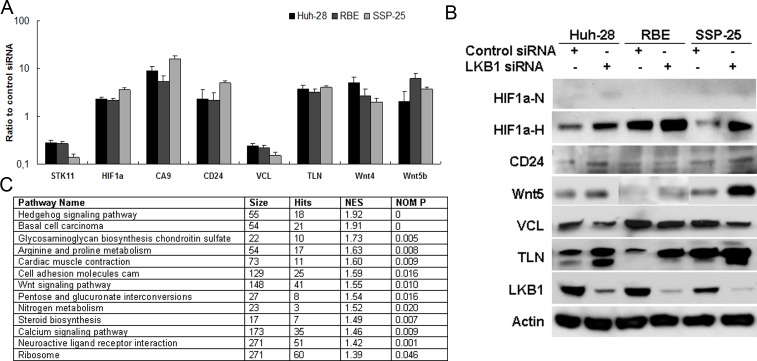
Identification of gene targets and signaling pathways of LKB1 inactivation in ICC by RNAseq analysis and gene set enrichment analysis Candidates identified by RNAseq analysis of LKB1 siRNA versus control transfected ICC cell lines were validated by **A.** qRT-PCR analysis and **B.** Western-blot analysis. qRT-PCR is presented as ratio/fold difference of LKB1 siRNA transfected to control siRNA transfected cells. HIF-1α protein was measured under both normoxia (HIF1α-N, 21% O_2_) and hypoxia (HIF1α-H, 1% O_2_ for 18h). **C.** The list of gene set enriched by knockdown of LKB1 in ICC cells.

To determine the effects of *LKB1* knockdown on global gene expression in three ICC cell lines (HuH-28, RBE, and SSP-25), used small interfering RNA (siRNA), RNAseq and quantitative pathway analysis. About 212 genes differentially expressed between the *LKB1* knockdown and control cells in the three cell lines (Supplementary Table 3), are also shown in the heat map (Supplementary Figure 4). Interestingly, the RNA sequencing data analysis also uncovered a variety of genes that were significantly dysregulated in all three ICC cell lines with attenuated LKB1, such as *HIF-1α, CA9, CD24, Talin1 (TLN1), Vinculin (VCL)*, and *Wnt4/5* that participate in hypoxia response, cell adhesion, migration, and metastasis. Differential gene expression of LKB1, HIF-1α, CA9, CD24, VCL, TLN1, Wnt4, and Wnt5 were confirmed by qRT-PCR (Figure 4A) and Western blot (Figure 4B). Using GSEA [26], we identified multiple gene sets from the Kyoto Encyclopedia of Genes and Genomes (KEGG) that were significantly enriched in LKB1-attenuated ICC cells (*P* < 0.05). The top commonly enriched gene sets in these three cells were Hedgehog signaling, Wnt/β-catenin signaling, and cell adhesion and arginine/proline metabolism (Figure 4C).

### Enhancement of Wnt/β-catenin pathway in ICC following LKB1 knockdown

**Figure 5:**
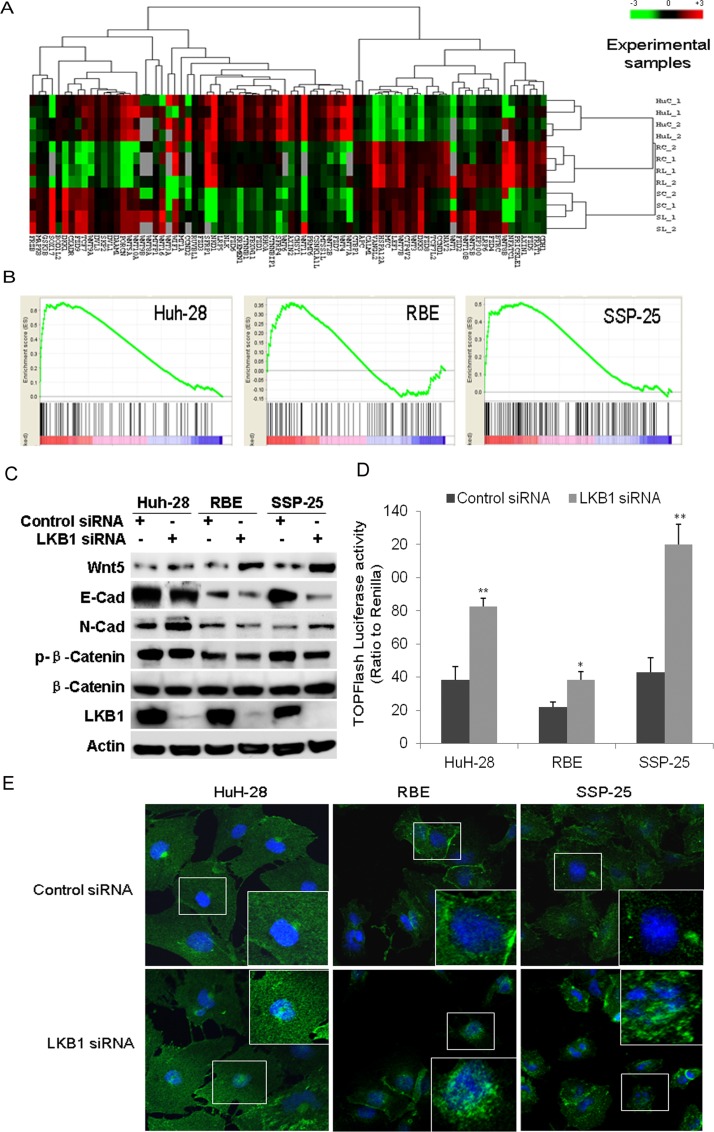
GSEA identified the enrichment of Wnt/β-catenin upon knockdown of LKB1 in ICC cells **A.** Heat map of relative enrichment scores for Wnt signaling pathway genes in siRNA transfected ICC cells as indicated. Gene signatures are represented in rows (red = significant enrichment of overexpressed genes; green, significant enrichment of under-expressed genes; black, not significant; *P* < 0.05). ICC cells and conditions are represented in columns (HuC: HuH-28 transfected with control siRNA, HuL: HuH-28 with LKB1 siRNA, RC: RBE with control siRNA, RL: RBE with LKB1 siRNA, SC: SSP-25 transfected with control siRNA, SL: SSP-25 with LKB1 siRNA, 1 for the 1^st^ test, 2 for the 2^nd^ test. **B.** Enrichment plots of the Wnt/β-catenin gene set in all three LKB1-attenuated ICC lines. **C.** Western-blot analysis to validate selected proteins involved in Wnt/β-catenin signaling and EMT markers. **D.** Quantitation of TOPFlash luciferase activity of TCF promoter reporter in ICC cells transfected with control or LKB1 siRNA as indicated (**P < 0.01, compared to control siRNA). **E.** Double-label fluorescent immunohistochemistry of cells as indicated at 72h posttransfection with LKB1 or control siRNA in the three ICC cells. Blue is DAPI nuclear stain and green is β–catenin. Inserts show magnified areas.

We next investigated the effect of *LKB1* knockdown on the Wnt/β-catenin pathway in ICC cells. On the heat map displaying 141 genes in the Wnt/β-catenin pathway, we found dramatic change of expression of 41 genes after knockdown of LKB1 in the three ICC cell lines (Figure 5A). GSEA analysis showed that the Wnt/β-catenin signaling pathway was significantly enriched in the three LKB1-attenuated ICC cell lines (Figure 5B, *P* < 0.01). Interestingly, several ligands such as Wnt2b, Wnt4, Wnt5b, and several receptors such as the frizzled gene family members FZD1/2/7 were uniformly upregulated in all three LKB1-attenuated ICC cells. Surprisingly, expression of negative regulators of the Wnt signaling pathway such CDH1 was also significantly decreased in LKB1-attenuated ICC cells. qRT-PCR analysis showed that the expression of Wnt target genes such as *CYCD1, C-MYC, FZD2* was significantly upregulated upon LKB1 knockdown in these three ICC cell lines (Supplementary Figure 4). Western-blot further showed that Wnt-5B protein was upregulated, while E-Cadherin is significantly downregulated, and N-Cad was significantly upregulated in all three ICC cells (Figure 5C, *P* < 0.01). Serine-phosphorylation of β-catenin targets it for ubiquitination-degradation [27], and it is notable that we saw a dramatic decrease in phosphorylated β-catenin in all three LKB1-attenuated ICC cell lines, while total β-catenin was not altered (Figure 5C). All the above data strongly point to activation of Wnt/β-catenin upon LKB1 suppression.

To further examine the activation of Wnt and nuclear accumulation of β-catenin by LKB1 knockdown, we measured T cell factor (TCF) transcriptional activity using TOPFlash-Luc (Millipore), as a reporter plasmid [27]. As β-catenin is an obligatory cofactor for TCF, increase in TCF transcriptional activity indicates upregulation of β-catenin. Knockdown of LKB1 in all three cell lines significantly activated TCF transcriptional activity (Figure 5D). Consistently, overexpression of LKB1 substantially suppressed the activity of TOPFlash-Luc reporter and increased phosphorylated β-catenin in these three cell lines (Supplementary Figure 5). Moreover, we further examined whether LKB1 knockdown affected the nuclear accumulation of β-catenin. Double-label fluorescent immunohistochemical analysis showed that knockdown of LKB1 induced the accumulation of nuclear β-catenin in all three ICC cells (Figure 5E). These results suggest that LKB1 suppression induces nuclear localization of β-catenin and concurrent TCF activation.

### The inverse correlation between LKB1 and β-catenin predicts aggressive clinicopathological characteristics and poor prognosis in ICC patients

**Figure 6:**
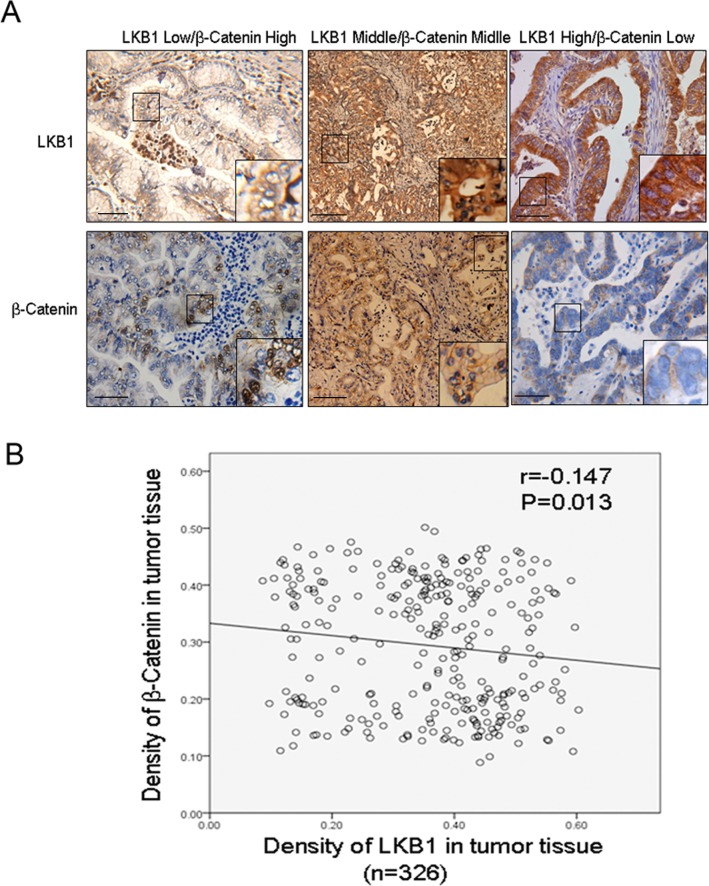
Inverse correlation between LKB1 and nuclear β-catenin in ICC tissues **A.** Representative IHC staining of LKB1 and β-catenin (x200) in serial sections, scale bar: 20 μM. Shown are low LKB1 (left), moderate LKB1 (middle) and high LKB1 (right). Inserts indicate magnified areas. **B.** Regression plot of LKB1 and nuclear β-catenin in 326 patient tissues, analyzed by Spearman's nonparametric correlation test (R = −0.147; *P* < 0.05).

Our *in vitro* cellular study demonstrated activation of Wnt/beta-catenin upon LKB1 inactivation. We next studied the correlation between LKB1 and nuclear β-catenin in 326 ICC tissues. Figure 6A shows representative IHC staining of LKB1 and β-catenin.. Using Spearman's nonparametric correlation analysis we identified an inverse correlation between LKB1 and nuclear β-catenin (Figure 6B, R = −0.147, *P* < 0.05). This further indicates that upregulation of Wnt/β-catenin signaling following LKB1-inactivation contributes to carcinogenesis and progression in ICC tissues.

## DISCUSSION

Our current study represents the first investigation of the important role of LKB1 underexpression in a large ICC cohort. We have for the first time identified LKB1 underexpression was inversely associated with malignancy and poor survival of ICC patients, and suggested a novel mechanism for ICC carcinogenesis in which LKB1 underexpression enhances multiple signaling pathways including Wnt/β-catenin to promote disease progression.

In this study, we first found *LKB1* gene deletions and missense mutations including the well-characterized loss-of-function mutation of c.C1062G, p.F354L in ICC tissue samples [24], albeit in low frequency. These genetic alterations lead to complete inactivation or haploinsufficiency of *LKB1* tumor suppressor in ICC tissues. Although hypermethylation of *LKB1* gene was frequently found in pancreatic carcinoma [28], our pyrosequencing analysis revealed that hypermethylation was not a frequent event in ICC tissues. Although the frequency of genetic alterations leading to LKB1 loss or underexpression was not high, immunohistochemical analysis showed that LKB1 protein expression was reduced in a moderate part of ICC tissues compared to adjacent tissues. This discrepancy implies other mechanisms such as undiscovered mutations in promoter and splicing regions of LKB1 or dysregulation of miRNAs are responsible [29-31]. Moreover, we showed that LKB1 protein was inversely correlated with malignancy and survival of ICC patients. This finding in our clinical cohort is consistent with reports of LKB1 deficiency significantly enhancing the metastasis of activated oncogenic K-Ras-driven lung adenocarcinomas and melanoma in mice [21, 22].

In this study, we, using RNAseq data analysis, uncovered a variety of proteins participating in cell adhesion, migration and metastasis such as *TLN1, VCL* that were modified upon loss/downregulation of LKB1 in ICC cells. For example, *TLN1*, a cytoskeletal protein that was upregulated in LKB1-attenuated ICCs is directly correlated with invasion and metastasis of human oral squamous cell carcinoma [32]. Vinculin, an adhesion protein that participates in cell-cell adhesions and that is inversely correlated with the metastatic potential of human squamous cell carcinomas [33], was significantly downregulated in all LKB1-attenuated ICCs. These two results indicate that perturbed cell adhesion molecular signaling might contribute to the malignancy seen in LKB1-downregulated ICC cells. Additionally, we also found that HIF-1α is upregulated upon LKB1 knockdown, and this is consistent with a recent report that LKB1-dependent reprogramming of cell metabolism is dependent on HIF-1α, and that transcription of HIF-1α is also upregulated in LKB1-deficient cells [23]. Increased expression of the cancer stem cell-associated membrane protein CD24 correlates with tumor aggressiveness; it is also a direct target of HIF-1α and participates in tumor growth and metastasis [34]. Interestingly, another expression profiling study of human lung cancer cell lines and mouse lung tumors identified a variety of metastasis-promoting genes, such as *VEGF, CD24*, and *NEDD9* as targets of LKB1 repression in lung cancer [21].

Using GSEA analysis we identified multiple gene sets including Hedgehog signaling, Wnt/β-catenin signaling, and cell adhesion and arginine and proline metabolism that were significantly enriched in LKB1-attenuated ICC cells. Indeed, it has been shown that LKB1 was a critical mediator in both the Hedgehog and Wnt pathways in murine cultured cells [35]. Deregulation of Wnt/β-catenin signaling is associated with pathogenesis of several human malignancies. Since the cross-talk between the Wnt/β-catenin and LKB1 pathways is still largely unknown, we undertook to investigate the effect of LKB1 knockdown on expression of genes in the Wnt/β-catenin pathway. We found multiple Wnt ligands such as Wnt2b, Wnt5, Wnt10 and several receptors including the frizzled gene family members FZD1/2/7 were uniformly upregulated in all three LKB1-attenuated ICC lines. Interestingly, the protumorigenic role of Wnt5 enhancing migration and invasion of tumor cells has been shown in melanoma and gastric cancer [36]. β-catenin is an obligatory transcriptional coactivator for the TCF family of transcriptional activators of Wnt-responsive genes. In the absence of Wnt stimulation, serine-phosphorylation of β-catenin by GSK-3β targets it for ubiquitination-degradation, thus blocking transcriptional activation of the Wnt-responsive genes [37]. In all three LKB1-attenuated ICC cell lines we observed a dramatic decrease in phosphorylated β-catenin together with an increase in TOPFlash luciferase reporter activity, while total β-catenin levels were unaltered. This indicates a potential mechanism whereby loss or decrease in LKB1 levels upregulate Wnt family gene expression.

The E/N-cadherin switch during epithelial-mesenchymal transition (EMT) is significantly associated with poor prognosis in cholangiocarcinoma [38], and LKB1 is involved in EMT of human lung cancer cells [21, 22]. Consistent with this, we observed significant downregulation of both E-cadherin mRNA and protein, while N-cadherin was significantly upregulated in LKB1-attenuated HUH-28 and SSP-25 ICC cells. E-cadherin, a negative regulator of the Wnt signaling pathway, has been shown to recruit β-catenin to the cell membrane to prevent its nuclear localization and thus transactivation of Wnt-responsive genes [39]. In addition, disruption of the membranous distribution of β-catenin and E-cadherin has been implicated in the invasion and metastasis of intraductal papillary neoplasm of the bile duct [40]. Although we only focused on the canonical Wnt pathway, we cannot exclude the activation of non-canonical Wnt signalling upon LKB1 loss. We observed that some non-canonical ligands such as Wnt-5a were also significantly upregulated by LKB1-knockdown in ICC cells. In support of a role for the non-canonical pathway in LKB1 loss Wnt5a expression has recently been shown to be elevated in polyp formation in Lkb1+/− mice and Peutz-Jeghers syndrome [41]. In addition Wnt5a enhances migration and invasion of tumor cells in melanoma and gastric cancer models [36, 42]. Also, it has been proposed that the activation of the Wnt/β-catenin pathway in intraepithelial neoplasia in Lkb1−/− mice involves the inactivation of Gsk3β complex by Par1A [43]. LKB1 has been recently shown to regulate the Wnt pathway through a direct interaction with APC to suppress the tumorigenic/metastatic potential of lung tumors [44]. In the present study, we also identified an inverse correlation between LKB1 and nuclear β-catenin in the large ICC cohort. Considering the relatively large sample size and small R2, the clinical relevance for the correlation may be cautiously interpreted. We consider that one of the major reasons is that there are multiple factors or pathways contribute to over-activation of Wnt signaling in ICC. And underexpression of LKB1 partially enhances Wnt signaling in ICC.

In this study, using a transcriptional approach, we have proposed a complicated activation of Wnt signaling at various levels upon loss of Lkb1 function in ICC cells. Therefore, inactivation or downregulation of LKB1 may be a novel mechanism that promotes nuclear relocation of β-catenin in ICC tissues. Additionally, we have only focused on the Wnt/ β-catenin pathway in this study. However, other important signaling cascades such as cell adhesion, or arginine and proline metabolism may also be engaged in the enhanced malignancy in ICC upon LKB1 inactivation. These signaling cascades should be further examined in the future. Especially, the underlying mechanisms for these correlations could provide insights into potential therapeutic approaches.

## MATERIALS AND METHODS

### Patients selection and clinical data collection

The study was reviewed and approved by the Institutional Review Board (IRB) of Eastern Hepatobiliary Surgery Hospital (Shanghai, China). A total of 326 Chinese patients with pathologically diagnosed ICC who received primary and curative surgical operations between 2002 and 2010 were recruited. The details of their demographic and survival data are summarized in Supplementary Table 1. The patients were followed up every 2–4 months after discharge and were monitored prospectively by CA 19-9, CEA, and liver function tests, and abdominal ultrasound. Contrast-enhanced CT or magnetic resonance imaging was performed once every 6 months or sooner when tumor recurrence or metastasis was suspected. Recurrence/metastasis was defined by the presence of new tumors as detected and conﬁrmed by two radiologic methods. Overall survival (OS) was deﬁned as the interval between primary surgery and death or the last date of follow-up. Time to recurrence (TTR) was calculated from surgery to the date when recurrence/metastasis was diagnosed. TTR or OS was censored at last follow-up (Oct 18, 2012) for the patients without recurrence or death.

### Antibodies, western blot, and immunohistochemistry analysis

The rabbit polyclonal anti-LKB1 antibody (ab58786, Abcam) and rabbit monoclonal anti-β-catenin antibody (MA1-300, Thermo Scientific) were used for IHC analysis. The mouse monoclonal antibodies against β-actin were purchased from Sigma-Aldrich. The mouse monoclonal antibodies against Vinculin, and rabbit polyclonal against Talin 1 were purchased from Santa Cruz Biotechnology (Santa Cruz, CA, USA). The rabbit monoclonal antibodies against AMPK-alpha and phospho-AMPK (Thr172), 4EBP1, phospho-4EBP1 (Thr70), S6K, phospho-S6k (Ser371), E-Cadherin, N-Cadherin, phospho-β-Catenin, HIF-1α, and Wnt-5a/b were purchased from Cell Signaling Technology (Beverly, MA USA). The mouse and rabbit monoclonal antibodies against CD24 were purchased from Thermo Fisher Scientific Inc. (Rockford, IL, USA). Quantitative analysis of IHC staining were performed by scanning the slides with an Aperio ScanScope GL, and the Aperio ImageScope software (Aperio Technologies, Vista, CA) was used to assess the scanned images based on the percentage of positively stained cells and staining intensity. Expression levels of LKB1 and β-catenin in all clinical samples were quantiﬁed, and the ratio of LKB1 and β-catenin expression level between each pair tumor/peritumor was calculated as described previously [45].

### Fluorescence *in situ* hybridization (FISH) assay

Dual-color FISH was performed using the FISH-mapped confirmed the bacterial artificial chromosome (BAC) probes; BAC clones RP11-81m8 (chromosome 19p13.3) for LKB1 labeled in spectrum orange (Abbott Laboratories) and RP11-283b8 (chromosome 19q13.2) labeled in spectrum green (Abbott Laboratories) for a reference probe of chromosome 19 were selected for FISH assays. FISH assays were done on unstained FFPE slides from human ICC tissues according to the method we previously reported [46]. Two red and two green signals were considered normal in a diploid genome. Due to truncation and overlapping cells, at least 10% of 200 interphase cells must show an abnormal pattern to be considered abnormal.

### Pyrosequencing assay

The methylation levels of eight 5′-CpG islands in the previously defined region of *LKB1* gene (+301-+423; XM_005259617) [47] were analyzed by pyrosequencing analysis using the PyroMark kit (Qiagen). Genomic DNA from 16 ICC tumors and nonmalignant tissues specimens was treated with sodium bisulfite using EpiTect Bisulfite kits according to the manufacturer's instructions (Qiagen, USA). The PCR amplification was carried out by sense primer: 5′-GGAGGATGATTTAGTATTGAAAAGTT-3′ and biotinylated antisense primer 5′-TCCCACTTCCCTTCTCCAAAATTTTAC-3′ for 45 cycles of 95°C for 15 sec, 56°C for 15 sec and 72°C for 30 sec. The sense primer AGGTTTAGGGTTTTATTGGAAT (+361) was used to measure the degrees of methylation at 8 CpG sites over 70 bases. The amount of C relative to the sum of the amounts of C and T at each CpG site after bisulfite conversion was calculated as a percentage (%). An overall LKB1 methylation level is calculated as the average of the proportion of C (%) at the 8 CpG sites.

### LKB1 knockdown, overexpression, cellular proliferation, migration and invasion assays

ICC cell lines HuH-28, RBE, and SSP-25 were newly purchased from RIKEN Bio Resource Center (Tsukuba, Japan, http://www.brc.riken.jp/lab/cell/), and were newly DNA fingerprinted to confirm identity as previously described [48]. The expression of competent LKB in these three cells were confirmed by FISH (Supplementary Figure 1A-1C), RNA sequencing, and functional assays, which was demonstrated by increased phospho-AMPK upon the stimulation of metformin, a mitochondrial inhibitor (Supplementary Figure 1D). Cells were transfected with either scramble siRNA or LKB1-specific siRNA from Life Technologies and Santa Cruz Biotechnology, Inc. (Santa Cruz, CA) as what we previously described [46]. Proliferation, migration, invasion assays were also performed as what we described previously [49].

To establish stable SSP-25 cells with attenuated and overexpressed LKB1 protein, SSP-25 cells were first transfected with control or LKB1 gene-specific shRNA plasmids or pcDNA3.1/Zeo(+) expressing the open-reading frame of human LKB1 gene, and selected by medium containing 0.5 μg/mL puromycin or 0.25 μg/mL zeocin. Resistant colonies were obtained after puromycin selection. Substantial knockdown of LKB1 protein in these colonies was confirmed by Western blot analysis compared with the control shRNA transfected SSP-25 cells. The animal protocol for tumorigenicity assay *in vivo* was approved by the Institutional Animal Care and Use Committee of Taipei Medical University. Subcutaneous xenograft tumors were generated and analyzed in 6 to 8-week-old Nod-Scid mice (T) according to the method we previously described [49]. Six to 8-week-old Nod-Scid mice (T) were subcutaneously inoculated in the right flank with either 1 × 10^7^ SSP-25-control (expressing control shRNA) and SSP-25-KD1/2 cell lines (expressing LKB1-shRNA1/2 to substantially attenuate LKB1 protein expression) resuspended in 100 μL serum-free RPMI.

### RNA sequencing and LKB1 exon sequencing analysis

At 48h post-transfection of siRNA, total RNA was extracted for RNA sequencing analysis. Transcriptome libraries were constructed with TruSeq RNA Sample Preparation Kit V2 (Illumia, San Diego, USA) with minor modifications. In brief, 500 ng of total RNA from each sample was used to construct cDNA library, and then were sequenced on the Illumina Hiseq 2500 with single read 40 bp reads following the manufacturer's recommendations. High-quality reads were aligned to the human reference genome (NCBI Build 36.1) using NextGENe^®^ software (Softgenetics, State College, PA, USA). The matched reads were aligned to Human Refseq mRNA (NCBI). A mean log_2_ fold change [LKB1-attenuated cells/control cells transfected with scramble siRNA] of each gene was calculated across all 3 cell lines. The false discovery rate (FDR, i.e. a probability of wrongly accepting a difference between cells with LKB1 knockdown and parental cells) of each gene was determined according to the method previously reported [50]. The genes were regarded as differentially expressed when their FDRs were less than 0.05. Further, genes were classified as up-regulated when their mean log_2_ fold change ratio was larger than 1 or down-regulated when their log_2_ fold change ratio was less than −1. Gene set enrichment analysis was applied to examine pathways significantly modulated by knockdown of LKB [26]. All coding exons of LKB1 were amplified and sequenced by Sanger sequencing. Primers' sequence will be provided upon request. The genomic DNA samples were not extracted from cancer cells procedured by tissue microdissection but formalin-fixed, paraffin-embedded (FFPE) tissues, therefore, a DNA sequencing pattern for a specific mutation identified in a sample may be heterozygous, except it is a germline mutation that was presented as homozygous. The sequence was aligned to Human Refseq (NCBI). If at least 5% of the cells (based on peak of mutation) in a tissue harbored a mutation, the sample was assessed as abnormal or mutated.

### Dual luciferase reporter assay

ICC cells were seeded at a concentration of 5.0 × 10^4^ cells per well on 24-well plates. Cells were transfected with 125 ng of T-cell factor (TCF)/Lef-1 reporter plasmid plus 5 ng *Renilla* luciferase reporters using Lipofeactamine™-2000. Forty-eight hours after reporter plasmid transfection, firefly and *Renilla* luciferase activities were determined according to the manufacturer's instruction for the dual luciferase assay (Promega, Madison, WI). Relative luciferase activity of each reporter was normalized to the value of Renilla luminescence. All experiments were done in triplicate. Data were reported as means ± standard error.

### Immunofluorescence

Subconfluent cells cultured on gelatin-coated glass coverslips were fixed 15 min in 4% paraformaldehyde in phosphate-buffered saline (PBS), permeabilized in 0.15% Triton-X100 in PBS and blocked in 1% BSA, 0.05% Tween-20 in PBS. The rabbit monoclonal antibody against β-catenin (Thermo Scientific, 1:500 dilution) was incubated overnight at 4°C followed by visualization using secondary antibody and Vectashield (Vector Laboratories) containing 4′,6-diamidino-2-phenylindole 1:1000 (Invitrogen). Immunofluorescent images were taken using Zeiss LSM510 upright confocal microscope.

### Data management and statistical analysis

The JMP 11.1 Software (SAS Institute) was used for statistical analysis. Group comparisons for continuous data were conducted using Student's *t* tests or χ^2^ tests, and for quantitative variables were analyzed with the paired the Wilcoxon signed-rank test or the Spearman rank correlation test. Kaplan-Meier analysis was used to assess survival. Log-rank tests were used to compare patient overall survival (OS) and time to tumor recurrence (TTR) between subgroups. The Cox hazard proportional model was applied to multivariate analysis. The results of the cell function experiments were presented as the means ± standard error. Statistical significance was set at *P* < 0.05.

## SUPPLEMENTARY FIGURES AND TABLES


