# Supplementary material for: Underexpression of LKB1 tumor suppressor is associated with enhanced Wnt signaling and malignant characteristics of human intrahepatic cholangiocarcinoma
Source: Oncotarget. 2015 May 27;6(22):18905–20. doi: 10.18632/oncotarget.4305 (PMC4662463; doi:10.18632/oncotarget.4305)
Supplement: Supplementary file 1 [file oncotarget-06-18905-s001.pdf]

Underexpression of LKB1 tumor suppressor is associated with enhanced Wnt signaling and malignant characteristics of human intrahepatic cholangiocarcinoma

Supplementary Material

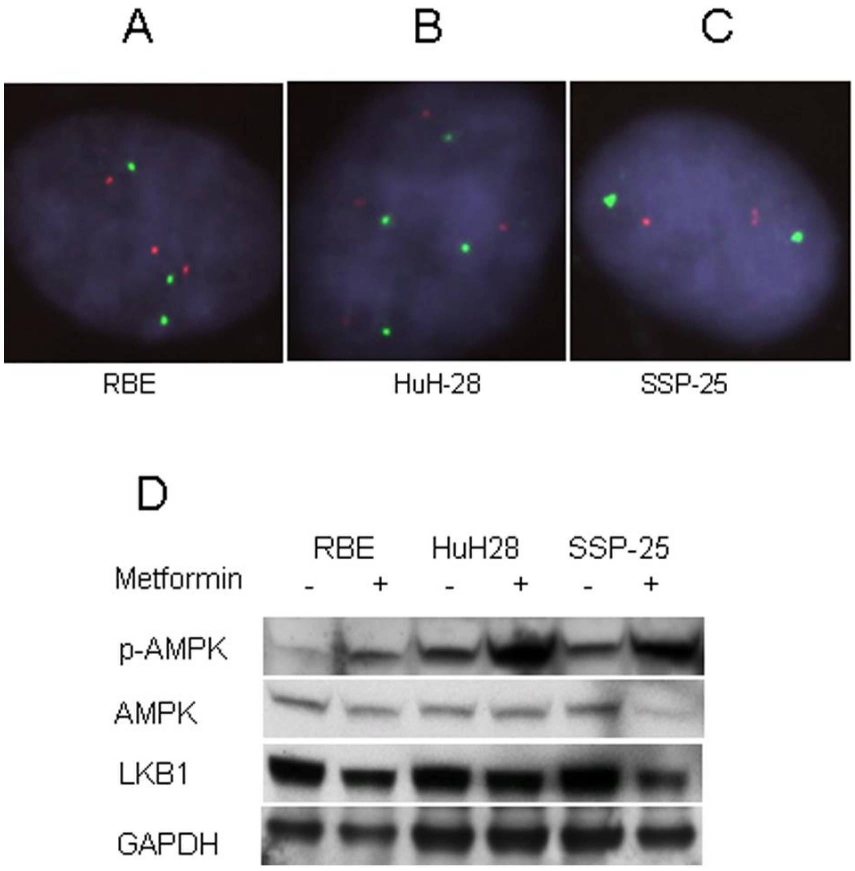

Supplementary Figure. 1. Dual-color fluorescence in situ hybridization (FISH) of the three ICC cells. RBE cells (**A**) have trisomy of LKB1 at chromosome-19. HuH-28 (**B**) and SSP-25 (**C**) harbor normal diploid of LKB1 manifesting by 2R/2G (two red spots for LKB, two green spots for chromosome 19 centromere). **D.** Metformin-induced AMPK activation in three ICC cells. Western blot showed the basic and metformin-induced (+: 25 mM metformin for 24h) p-AMPK (phosphorylated AMPK-α at Thr172) in three ICC cell lines. The same blotted membrane was developed for p-AMPK, LKB1 and then consecutively re-probed for AMPK and GAPDH.

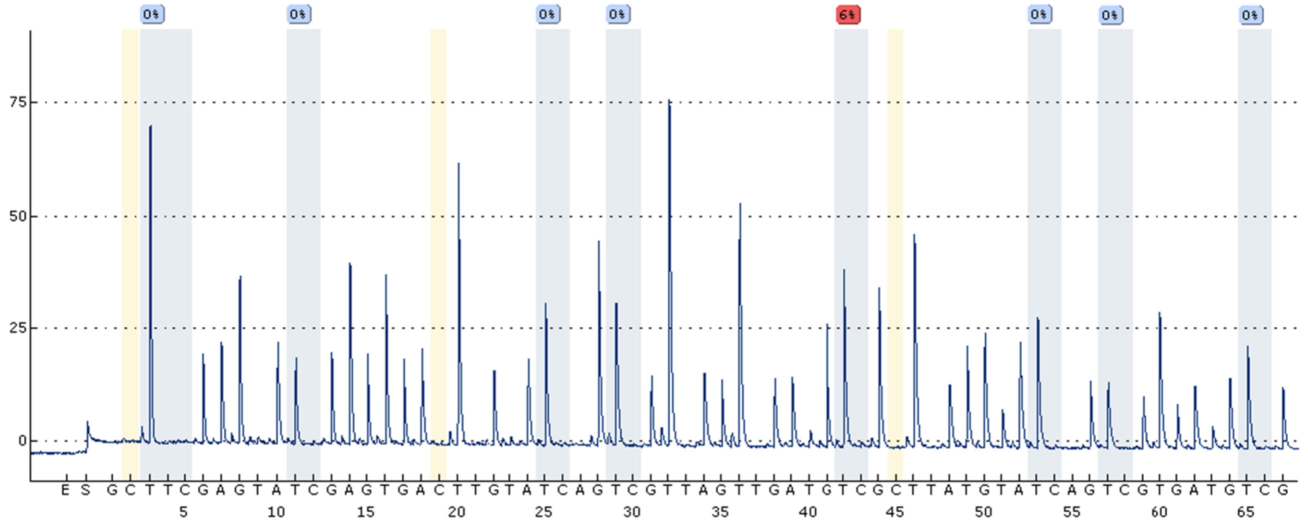

Supplementary Figure. 2 Measurement of the LKB1 methylation level using pyrosequencing in a representative non-malignant tissue.

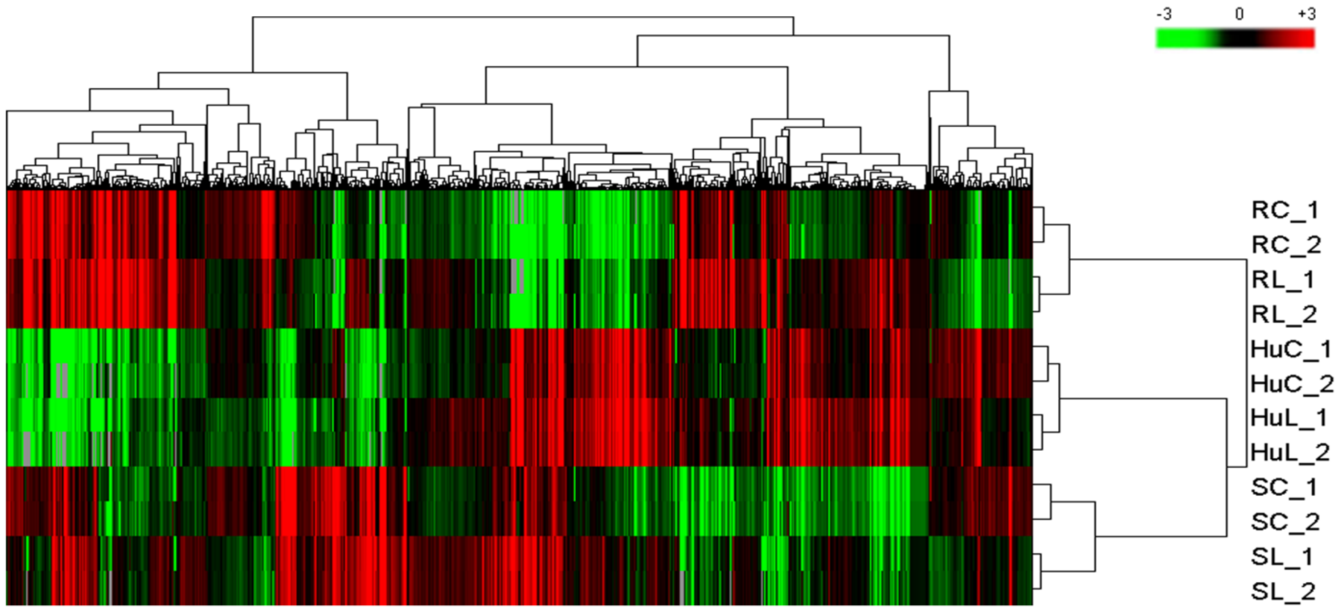

Supplementary Figure 3. Unsupervised clustering analysis heatmap of 329 gene signatures upon LKB1 knockdown in three ICC cells. Each colored square on the heatmap represents the relative signature score (in log2 space) for each sample with highest expression being red, lowest expression being green and average expression being black.

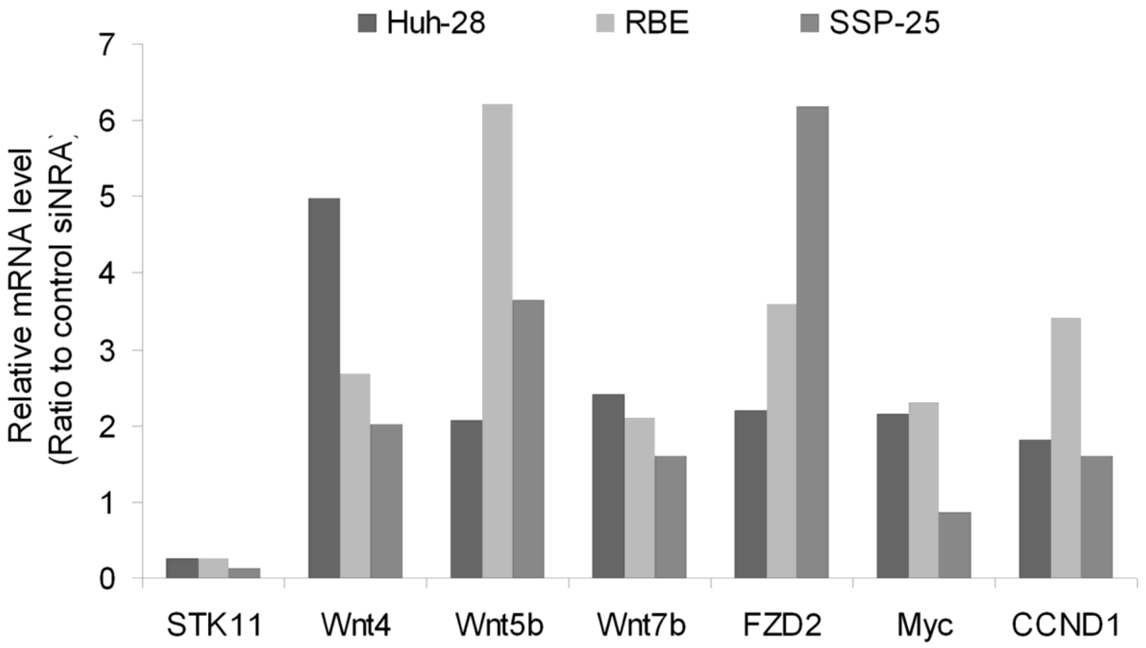

Supplementary Figure 4. Histogram of qRT-PCR validation results for differentially expressed genes involved in Wnt signaling in three ICC cell lines after LKB1 knockdown. Relative mRNA levels of selected genes in LKB1-attenuated ICC cell lines were presented as ratio to control cells transfected with scramble control siRNA.

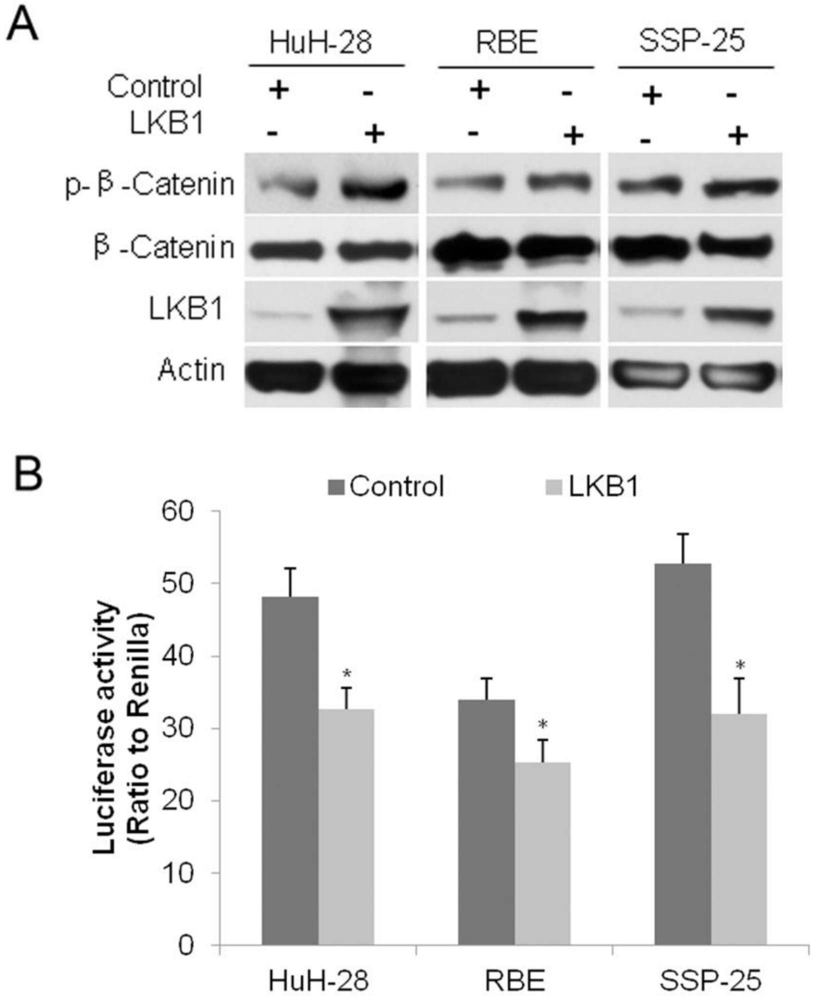

Supplementary Figure 5. Impact of overexpression of LKB1 in ICC cells on Wnt signaling pathway. **A.** Western-blot analysis of LKB1 and β-catenin in control (GFP-transfected) and LKB1-overexpressed ICC cells. **B.** Quantitation of TOPFlash luciferase activity of TCF promoter reporter in ICC cells transfected with control (GFP) or LKB1 plasmids as indicated (**P* < 0.01, compared to control GFP transfected)**.**

Supplementary Tables

Supplementary Table 1. Demographics and clinicopathologic features of patients with intrahepatic cholangiocarcinoma.

Parameter	No. of Cases	%
Age		
≤58	170	52.2
>58	156	47.8
Gender		
Male	214	66.0
Female	112	34.0
Alcohol intake		
Yes	174	53.4
No	152	46.6
CA19-9 level		
≤37 (U/ml)	135	41.4
>37 (U/ml)	191	58.6
Virus infection		
HBV	153	46.9
HCV	19	5.8
HBV+HCV	8	2.4
None	146	44.8
Liver cirrhosis		
No	252	77.3
Yes	74	22.7
Tumor size		
≤5cm	151	46.3
>5cm	175	53.7
Differentiation (a)		
Poor	71	21.8
Moderate	181	55.5
Well	74	22.7
Vascular invasion		
No	202	61.7
Yes	134	38.3
Lymph Node		
No	208	63.8
Yes	118	36.2
Encapsulation		
Complete	36	11.1
None	290	88.9
Intrahepatic metastasis		
No	239	73.3
Yes	87	26.7
TNM stage (b)		

I-II	173	53.1
III-IVA	153	46.9
Recurrence		
No	113	34.7
Yes	213	65.3

Note: TNM, tumor–node–metastasis; (a) Tumor differentiation was determined according to the "British Society of Gastroenterology guidelines on the management of Cholangiocarcinoma; (b) American Joint Committee on Cancer 7th edition staging for intrahepatic Cholangiocarcinoma.

Supplementary Table 2. Mean CpG methylation (%) of 8 consecutive CpG sites (labeled Pos 1-8) in ICC and non-malignant tissues.

CpG methylation of each site	Pos 1	Pos 2	Pos 3	Pos 4	Pos 5	Pos 6	Pos 7	Pos 8	Overall methylation
ICC (N=21)	2.6	1.5	5.6	1.4	10.8	11.4	1.9	4.0	4.9±3.8*
Non malignant tissues (N=12)	0	0	2.3	0	4.3	4. 7	0	0	1.4±1.9

* $p < 0.05$, the overall methylation levels of 8 CpG sites in ICC tissues compared with that in non-malignant tissues.

Supplementary Table 3. Differentially expressed genes in three LKB1-attenuated ICC cells

	HuH28-LKB1 siRNA vs. HuH28 Control siRNA		RBE-LKB1 siRNA vs. RBE Control siRNA		SSP25-LKB1 siRNA vs. SSP25-Control siRNA	
GeneName	log2FC*	P.Value	log2FC*	P.Value	log2FC*	P.Value
CEND1	3.63	1.02E-15	2.25	1.10E-01	5.45	1.82E-25
CA9	3.17	6.26E-11	2.41	7.74E-05	4.00	3.84E-05
NOVA2	2.97	4.26E-06	2.27	6.27E-02	2.22	3.96E-03
PGF	2.86	6.35E-04	1.76	1.22E-04	2.54	7.61E-13
ARSI	2.84	7.59E-03	1.56	3.39E-130	1.99	1.72E-89
NKAIN1	2.79	4.53E-06	2.92	8.01E-05	2.62	8.18E-11
PPP2R2B	2.61	2.99E-06	2.08	1.20E-10	2.79	3.93E-12
CHPF	2.51	8.64E-10	2.92	0.00E+00	3.20	0.00E+00
WNT4	2.34	1.47E-06	1.32	1.47E-01	1.01	4.21E-02
HIPK3	2.24	6.40E-08	2.19	6.16E-181	2.90	1.10E-275
PURG	2.21	1.47E-01	1.54	1.00E+00	2.78	1.19E-02
VGF	2.21	5.83E-08	1.98	2.82E-95	1.21	1.54E-03
SLC45A1	2.19	1.03E-04	2.78	3.05E-28	1.07	2.85E-08
C1orf94	2.19	3.83E-01	2.58	1.19E-08	1.10	1.00E+00
NEGR1	2.16	2.38E-08	3.00	2.01E-24	4.29	1.74E-190
EMP2	2.15	7.64E-08	2.11	2.64E-101	2.45	7.70E-98
ALCAM	2.09	5.28E-08	2.18	2.53E-222	2.20	3.73E-276
SLCO2A1	2.04	2.14E-05	1.58	3.14E-02	2.99	1.21E-60
GDF11	1.99	5.36E-06	2.02	1.92E-116	2.87	1.45E-196
RASD2	1.94	3.81E-05	2.29	2.23E-05	1.55	2.67E-32
STEAP3	1.94	1.95E-07	3.01	0.00E+00	3.17	0.00E+00
TMPRSS6	1.94	8.30E-02	1.60	4.62E-04	1.13	6.88E-01
FAM171B	1.94	2.24E-04	1.04	2.41E-23	1.90	1.03E-55
PCDH1	1.94	4.23E-06	1.31	2.67E-103	2.09	3.37E-46
TLN1	1.89	2.08E-06	1.66	8.28E-177	2.00	3.74E-255
DHX40	1.88	2.83E-06	1.85	2.27E-174	1.76	2.62E-130
LSM14A	1.83	2.04E-06	1.97	2.95E-158	1.88	2.76E-149
TNC	1.83	4.19E-06	1.59	5.32E-182	2.50	0.00E+00
HS6ST1	1.80	6.22E-06	1.53	1.37E-75	1.55	6.44E-48
IL6ST	1.79	3.29E-06	1.96	1.04E-221	2.09	5.76E-238
LMTK3	1.78	5.79E-02	1.16	4.90E-04	2.77	3.90E-02
FGG	1.77	5.39E-01	1.03	6.55E-05	1.48	5.82E-01
YWHAH	1.77	5.55E-06	1.69	1.38E-163	1.76	2.89E-173
STAB1	1.76	7.66E-05	1.79	2.00E-07	1.46	1.19E-12
ACPL2	1.76	8.37E-04	1.55	5.19E-12	1.89	1.90E-13

ISM1	1.76	6.37E-01	1.63	3.90E-09	2.17	1.32E-02
CAV3	1.75	5.09E-01	1.51	3.14E-02	1.10	1.00E+00
MFAP2	1.73	1.00E+00	1.53	1.25E-01	1.93	3.97E-14
GPR162	1.73	1.00E+00	1.53	2.50E-01	3.65	8.94E-04
NUDT15	1.69	3.66E-06	1.97	2.27E-117	2.72	8.49E-207
ELOVL7	1.66	1.58E-04	1.68	2.15E-65	1.98	7.94E-12
C12orf68	1.63	1.44E-03	1.38	6.16E-03	1.12	6.44E-04
CORO1A	1.62	5.81E-02	1.07	3.63E-09	1.81	2.02E-11
DLGAP3	1.61	3.75E-04	1.00	1.47E-01	1.24	1.57E-02
ANO10	1.60	2.11E-05	2.27	9.84E-151	1.92	7.18E-145
IL10RB	1.60	8.74E-05	1.43	1.95E-72	1.69	1.65E-92
NT5DC2	1.59	4.44E-05	1.59	1.19E-103	1.57	2.04E-114
MSX1	1.58	4.94E-05	1.35	6.65E-30	1.84	3.12E-14
CKMT1B	1.57	3.70E-04	1.68	3.62E-35	2.31	2.08E-04
PLAT	1.57	4.66E-05	1.21	1.02E-40	1.72	9.22E-71
SV2A	1.56	7.97E-02	1.35	7.72E-62	1.72	5.75E-11
PANK3	1.55	7.91E-05	1.92	4.50E-163	2.29	9.86E-202
NAT8L	1.55	6.40E-04	1.49	8.58E-39	1.47	2.05E-27
CKMT1A	1.55	3.20E-04	1.56	3.94E-34	1.77	5.49E-04
SNRNP27	1.52	1.63E-04	1.89	3.75E-81	2.05	4.27E-112
THG1L	1.50	1.61E-04	1.69	2.56E-53	1.57	2.14E-27
EHD3	1.50	1.01E-04	1.41	4.40E-21	2.16	2.44E-158
CISH	1.50	2.35E-03	1.68	7.04E-26	1.30	6.77E-06
CYB5R4	1.49	4.96E-05	1.09	5.38E-41	1.61	1.65E-73
CPLX1	1.48	1.99E-03	1.63	3.41E-30	1.50	1.47E-01
SLC11A1	1.48	4.11E-02	1.41	1.47E-03	2.40	6.30E-03
CHST15	1.43	2.30E-04	1.84	1.77E-143	1.35	4.73E-04
PTBP3	1.42	2.38E-04	2.09	3.78E-244	1.48	9.67E-121
IQSEC1	1.42	2.84E-04	1.80	2.94E-141	1.71	2.62E-68
SYT11	1.41	5.58E-04	1.78	1.16E-26	1.46	3.31E-04
FNDC5	1.40	5.53E-02	1.33	2.72E-02	1.01	3.72E-02
CLIC4	1.40	1.56E-04	1.46	2.91E-115	2.15	6.43E-262
CACHD1	1.39	1.94E-03	2.10	3.48E-25	2.84	2.52E-73
OSGIN2	1.38	2.36E-04	1.82	2.54E-124	1.88	2.76E-146
PROCR	1.38	1.39E-04	2.69	1.45E-48	1.52	5.61E-110
LIPG	1.34	1.51E-03	1.12	6.23E-64	1.13	2.82E-36
FAM135A	1.34	1.45E-03	1.60	1.79E-95	2.42	5.35E-104
SLC10A5	1.34	2.29E-01	1.02	1.41E-02	1.42	7.95E-02
SEPT4	1.33	2.07E-02	1.61	2.25E-04	1.15	1.89E-08
KIF5C	1.31	2.62E-04	2.22	1.52E-18	3.42	1.52E-78

SPOPL	1.31	3.42E-04	1.60	2.08E-63	2.02	4.39E-112
SLC41A1	1.31	1.20E-03	2.01	4.20E-184	2.08	1.93E-215
SAR1B	1.30	2.92E-04	1.49	3.45E-80	1.79	1.09E-125
CPNE2	1.30	1.57E-03	1.36	4.47E-52	1.54	6.24E-82
EDIL3	1.29	8.49E-04	2.57	3.39E-14	1.68	1.31E-117
GOLGA7B	1.28	3.00E-03	1.12	2.96E-52	1.29	3.25E-06
SLC24A4	1.28	6.28E-02	2.22	1.93E-04	1.16	2.68E-01
HAP1	1.27	5.04E-01	1.09	1.39E-05	1.90	6.47E-05
ZDHHC22	1.26	8.78E-04	1.93	1.57E-02	1.47	2.90E-01
TRABD2B	1.25	1.64E-03	1.23	2.94E-30	1.10	1.00E+00
FOXS1	1.25	7.34E-02	2.11	5.64E-04	3.44	1.24E-07
SPRED3	1.25	1.83E-03	1.13	1.16E-13	1.38	1.37E-07
MAPK3	1.24	4.18E-03	1.14	2.65E-47	1.54	2.01E-72
CHST2	1.24	4.06E-03	1.56	6.04E-28	1.00	1.34E-23
CDC14A	1.23	1.54E-02	1.10	2.52E-12	1.38	4.64E-25
RNF150	1.21	1.52E-02	1.46	4.61E-03	1.68	1.14E-05
SEC24A	1.21	2.71E-03	1.14	1.46E-52	1.35	1.05E-87
ZCCHC3	1.21	4.90E-03	1.43	2.41E-93	1.28	9.72E-69
SYNGR3	1.21	1.71E-04	2.46	5.69E-12	2.27	4.49E-07
CD24	1.20	2.60E-03	1.15	2.08E-63	2.33	3.25e-320
OLFML2A	1.20	3.09E-03	1.23	4.40E-45	1.34	1.97E-53
ATP11C	1.20	6.43E-03	1.28	1.75E-47	1.53	1.24E-28
B4GALNT4	1.18	7.03E-01	1.25	1.52E-53	1.46	4.79E-03
SOAT2	1.18	6.37E-01	3.08	3.08E-09	1.52	1.19E-02
ZNF469	1.18	6.37E-01	1.40	1.04E-82	1.54	4.85E-118
KRCC1	1.16	1.31E-03	1.20	8.63E-46	1.19	2.50E-26
TMEM41A	1.15	1.27E-03	1.73	2.11E-86	1.22	1.17E-54
PPP1R14B	1.15	2.68E-03	1.45	5.91E-125	1.03	4.22E-63
TOP1MT	1.15	8.19E-03	2.04	8.68E-113	1.89	2.73E-75
HHIP	1.15	3.54E-03	1.42	9.30E-02	2.18	9.75E-61
GDNF	1.14	1.15E-02	1.33	2.49E-02	1.59	2.21E-53
AVPR1A	1.14	1.00E+00	2.27	1.00E+00	2.78	3.31E-04
CSDC2	1.14	1.00E+00	1.94	6.27E-02	1.78	3.14E-02
ISM2	1.14	1.00E+00	2.52	7.89E-03	1.12	6.25E-01
PKP1	1.14	1.00E+00	1.85	1.35E-13	3.41	1.14E-05
ZFP57	1.14	1.00E+00	2.14	7.72E-15	1.71	5.00E-01
FAM83C	1.13	1.00E+00	2.67	3.89E-41	2.59	2.12E-04
PBX1	1.12	4.28E-03	1.03	8.81E-38	1.58	1.91E-60
HIF1A	1.12	2.35E-03	1.14	2.07E-78	1.24	1.09E-86
HERPUD2	1.12	4.26E-03	1.41	2.17E-63	1.08	6.13E-45

LUZP6	1.12	3.27E-03	1.50	2.98E-120	1.82	4.76E-193
MTPN	1.12	3.27E-03	1.50	2.98E-120	1.82	4.76E-193
MFSD6	1.12	4.70E-03	1.38	3.65E-37	1.46	1.09E-31
UCK1	1.11	6.63E-03	1.27	2.27E-57	1.47	1.75E-70
SNCB	1.11	4.80E-01	2.28	1.61E-21	1.71	5.00E-01
NTSR1	1.11	1.00E+00	1.95	6.88E-01	2.65	1.59E-03
VMO1	1.11	1.00E+00	2.06	1.35E-01	1.83	3.96E-03
TMEM63C	1.11	1.63E-01	3.26	2.51E-04	2.29	2.49E-08
NXPH4	1.11	1.30E-01	1.04	4.89E-02	1.25	5.44E-03
IRS2	1.10	7.44E-01	1.19	1.51E-82	1.49	3.30E-60
CSGALNACT1	1.09	6.72E-03	1.69	1.60E-27	1.74	8.99E-17
ANK1	1.09	9.52E-04	2.47	3.27E-119	1.26	8.45E-02
PPFIA4	1.09	4.66E-03	1.28	7.88E-09	1.58	2.87E-21
GLO1	1.08	5.42E-03	1.41	4.51E-109	1.09	2.36E-74
CCDC136	1.07	3.31E-02	1.07	1.54E-19	1.94	9.52E-57
CHST6	1.07	6.37E-01	1.33	1.67E-02	1.93	4.52E-08
FSCN2	1.07	7.95E-01	1.05	2.47E-06	1.15	6.25E-01
FAM216A	1.07	2.67E-03	1.20	2.47E-46	1.12	1.44E-38
SP6	1.07	1.18E-01	1.09	1.42E-04	1.10	3.33E-06
ZDHHC20	1.06	5.20E-03	1.54	4.82E-103	1.82	5.97E-158
SEC63	1.06	7.56E-03	1.37	2.56E-86	1.50	3.21E-123
FBXO41	1.06	1.56E-02	1.07	4.93E-49	1.51	1.15E-31
GOLGA4	1.06	7.98E-03	1.08	4.14E-65	1.51	3.87E-128
SS18L1	1.06	4.89E-03	1.25	3.10E-45	1.68	7.14E-86
ICOSLG	1.05	1.51E-02	1.21	3.70E-25	1.10	4.44E-15
PTP4A1	1.05	3.32E-03	1.23	1.66E-83	1.24	1.16E-90
CACNB1	1.05	1.05E-02	1.59	7.96E-33	1.21	5.59E-22
WNT5B	1.04	6.14E-03	2.63	6.40E-124	1.86	4.63E-103
PELI1	1.04	1.11E-02	1.36	3.34E-45	1.55	3.11E-29
ADAM19	1.03	8.59E-03	1.42	8.35E-30	1.50	2.05E-35
ANKRD34A	1.03	8.33E-04	1.16	1.21E-16	2.01	3.88E-26
FSD1	1.03	3.04E-02	2.11	3.20E-09	1.52	1.14E-19
ROBO1	1.03	1.02E-02	1.51	7.92E-108	1.72	3.50E-101
HSPA6	1.02	2.30E-01	1.61	8.42E-09	2.33	6.77E-28
CDH6	1.02	6.65E-03	1.76	6.32E-176	1.29	8.37E-29
RBM15B	1.02	7.49E-03	1.07	1.30E-71	1.23	2.70E-76
RBM38	1.02	6.24E-03	1.53	1.58E-78	1.51	1.60E-33
POLR3E	1.02	1.30E-02	1.25	1.47E-60	1.17	1.72E-47
DOCK9	1.01	1.04E-02	1.21	1.22E-81	1.66	3.22E-77
PAWR	1.01	7.30E-03	1.13	1.83E-62	1.02	1.96E-52

CACNG4	1.01	9.11E-02	2.28	5.14E-60	1.03	6.01E-21
CDH1	-1.02	9.54E-03	-0.87	2.98E-27	-1.17	6.95E-36
METAP2	-1.03	4.63E-03	-1.31	4.44E-110	-1.35	7.15E-120
WEE2	-1.05	7.44E-01	-1.44	1.69E-01	-1.21	2.32E-01
CDPF1	-1.06	2.59E-02	-1.46	2.63E-19	-1.94	1.54E-34
ESYT2	-1.06	1.02E-02	-1.54	1.53E-143	-1.69	2.49E-162
MRO	-1.07	1.00E+00	-1.64	1.91E-05	-2.84	9.96E-04
TGDS	-1.08	1.55E-02	-1.39	3.82E-32	-1.13	1.09E-32
AKAP6	-1.08	1.77E-02	-1.05	1.05E-15	-1.36	1.33E-14
IL18RAP	-1.09	6.37E-01	-1.53	1.00E+00	-2.03	6.27E-02
KLRC4	-1.10	3.96E-01	-2.95	3.05E-04	-1.41	5.20E-06
NLRP14	-1.11	5.09E-01	-2.92	1.47E-01	-1.04	6.30E-01
NLRX1	-1.11	3.33E-03	-1.20	2.95E-24	-1.15	1.67E-33
SGMS1	-1.12	1.08E-02	-1.10	4.31E-51	-1.14	2.45E-56
RAB11FIP2	-1.13	1.02E-02	-1.58	3.38E-94	-1.12	6.65E-44
HMMR	-1.15	6.81E-03	-1.62	9.14E-167	-1.29	2.52E-103
SPRYD3	-1.15	1.35E-03	-1.86	2.48E-138	-1.94	2.45E-176
GIMAP2	-1.19	1.16E-02	-1.93	1.25E-01	-1.08	1.55E-09
C8orf44-SGK3	-1.19	1.17E-03	-1.32	7.46E-31	-1.03	6.97E-24
FERMT2	-1.19	1.84E-03	-2.14	5.22E-163	-2.06	5.73E-222
SNAP23	-1.20	1.72E-03	-1.28	2.74E-70	-1.31	2.96E-108
SGK3	-1.21	4.54E-04	-1.40	5.05E-32	-1.14	2.33E-26
VPS36	-1.23	1.32E-03	-1.54	3.03E-124	-1.07	4.53E-51
RAB8A	-1.24	1.46E-03	-1.54	8.98E-112	-1.57	4.99E-117
VASN	-1.26	1.43E-03	-1.56	4.30E-94	-1.27	1.05E-32
DNAJC3	-1.26	2.14E-03	-1.35	1.71E-87	-1.10	1.26E-62
FLI1	-1.27	1.37E-03	-1.03	2.48E-14	-1.17	9.65E-61
NUS1	-1.28	2.02E-03	-1.56	3.46E-125	-1.32	3.24E-112
FBXL22	-1.31	1.33E-03	-1.30	7.05E-03	-1.01	4.74E-03
SCARB2	-1.31	6.93E-04	-1.31	2.48E-96	-1.69	4.06E-174
KCNMA1	-1.32	1.44E-02	-1.14	2.74E-45	-1.06	1.56E-10
CCDC102B	-1.32	2.89E-02	-1.07	7.79E-07	-1.69	1.73E-03
RTN3	-1.32	9.11E-04	-1.36	3.42E-126	-1.27	1.33E-109
AHCYL2	-1.34	1.83E-03	-1.31	1.01E-55	-1.09	1.57E-44
RPRD1A	-1.36	1.30E-03	-1.10	1.64E-66	-1.05	8.78E-41
PIP5K1A	-1.37	6.43E-04	-1.24	1.57E-89	-1.66	6.96E-150
FAR2	-1.38	8.23E-02	-1.15	2.59E-19	-1.25	8.57E-14
NRAS	-1.38	9.46E-04	-1.65	9.39E-176	-1.54	3.96E-159
PAFAH2	-1.39	9.59E-04	-1.44	1.31E-46	-1.62	3.89E-80
CLIP4	-1.39	7.25E-04	-1.23	6.69E-79	-1.12	9.74E-87

MARCH8	-1.44	5.47E-04	-1.02	8.93E-28	-1.22	2.66E-44
LPAR4	-1.61	5.00E-02	-2.31	4.16E-03	-1.62	1.32E-05
CCDC99	-1.62	4.84E-05	-1.77	1.52E-147	-2.00	7.65E-237
HOXC9	-1.63	1.00E+00	-1.51	1.25E-01	-1.03	1.53E-01
RAG2	-1.65	1.00E+00	-1.53	1.19E-02	-2.33	1.19E-02
RAB31	-1.66	3.06E-05	-1.75	3.84E-165	-1.77	6.53E-139
STK11	-1.66	4.59E-05	-1.93	5.68E-112	-2.88	5.48E-224
C4orf34	-1.67	1.34E-04	-2.00	3.60E-91	-1.99	1.68E-96
GBE1	-1.74	9.54E-06	-1.48	1.98E-89	-2.20	3.51E-227
PLCL1	-1.81	6.12E-02	-1.90	2.17E-02	-1.99	2.44E-03
GXYLT1	-1.84	6.97E-06	-1.87	7.78E-193	-2.10	1.21E-179
TTL	-1.87	3.01E-06	-1.79	1.48E-126	-1.82	3.10E-147
CLDN12	-1.89	1.33E-05	-1.57	1.14E-114	-2.11	5.00E-210
VCL	-2.07	7.49E-08	-2.23	0.00E-01	-2.75	0.00E+00
IRF4	-2.15	2.01E-02	-1.29	1.80E-01	-1.02	1.00E+00
LIPH	-3.26	1.00E-12	-1.96	3.88E-84	-3.96	2.74E-170
RPL21	-3.29	3.36E-06	-1.22	1.77E-25	-2.48	2.39E-33

*The mRNA expression levels of genes in LKB1-attenuated ICC cells are presented as log2 ratio to that of ICC cells transfected with control siRNA.
